# Techno-Economic Evaluation of Biodiesel Production from Waste Cooking Oil—A Case Study of Hong Kong

**DOI:** 10.3390/ijms16034362

**Published:** 2015-02-18

**Authors:** Sanjib Kumar Karmee, Raffel Dharma Patria, Carol Sze Ki Lin

**Affiliations:** 1 School of Energy and Environment, City University of Hong Kong, Tat Chee Avenue, Kowloon, Hong Kong; E-Mail: sanjibkarmee@gmail.com; 2 Department of Chemical and Biomolecular Engineering, the Hong Kong University of Science and Technology, Clear Water Bay, Kowloon, Hong Kong; E-Mail: rdpatria@ust.hk

**Keywords:** biodiesel, economic evaluation, equipment costs, capital investment costs, internal rate of return (IRR)

## Abstract

Fossil fuel shortage is a major challenge worldwide. Therefore, research is currently underway to investigate potential renewable energy sources. Biodiesel is one of the major renewable energy sources that can be obtained from oils and fats by transesterification. However, biodiesel obtained from vegetable oils as feedstock is expensive. Thus, an alternative and inexpensive feedstock such as waste cooking oil (WCO) can be used as feedstock for biodiesel production. In this project, techno-economic analyses were performed on the biodiesel production in Hong Kong using WCO as a feedstock. Three different catalysts such as acid, base, and lipase were evaluated for the biodiesel production from WCO. These economic analyses were then compared to determine the most cost-effective method for the biodiesel production. The internal rate of return (IRR) sensitivity analyses on the WCO price and biodiesel price variation are performed. Acid was found to be the most cost-effective catalyst for the biodiesel production; whereas, lipase was the most expensive catalyst for biodiesel production. In the IRR sensitivity analyses, the acid catalyst can also acquire acceptable IRR despite the variation of the WCO and biodiesel prices.

## 1. Introduction

The continuous increase in energy demand is contributing to petroleum-based oil depletion. In addition, inherent particle emitted by conventional fossil fuel combustion process has contributed towards the environmental pollution. Therefore, renewable energy sources are required for satisfying the energy demand. In this regard, biodiesel is considered a viable alternative fuel. As an energy source, biodiesel can reduce the emission level of the pollutants [1]. In addition, biodiesel is non-toxic and biodegradable, and it can be used along with conventional petroleum based fuels to create blends [2].

Commercially, biodiesel is produced using vegetable oils as feedstocks and chemical (acid/base) catalysts. Technically, it has been proven that the biodiesel production using vegetable oils as feedstock gives biodiesel with more than 90% yield [3]. Vegetable oils are costly. Therefore, biodiesel price using vegetable oil as feedstock (±$1.01/L) is expensive [4]. The oil feedstock contributes approximately 90% of the biodiesel production cost [5]. Therefore, an alternative feedstock is required for the cost-effective production of biodiesel.

In the above context, nonedible plant oils are used for the preparation of biodiesel [3,6,7]. Recently, lipid from food waste is used as a nonedible resource for biodiesel production [8,9]. Along this line, one of the alternative feedstock to produce biodiesel is waste cooking oil (WCO). WCO can be converted into biodiesel by transesterification [10,11,12]. Food supply will not be affected by the use of non-edible materials such as WCO as feedstock for biodiesel preparation. Therefore, the food *versus* fuel debate can be avoided. From the economics point of view, the use of WCO as a feedstock for biodiesel production is economically favorable ($0.36/L) [13]. Furthermore, it is estimated that 20,000 tons of WCO are generated in Hong Kong each year [14]. This data suggests that large quantities of waste cooking oil are available in Hong Kong. Along this line, WCO can be utilized as a beneficial and viable feedstock for the biodiesel production in Hong Kong.

In the above context, in this report economic analyses of the biodiesel production from WCO in Hong Kong with different catalysts are performed. Acid, base and lipase as the catalysts were evaluated for this techno-economic study. The techno-economic studies using these catalysts for biodiesel production processes were carried out using Aspen Plus as simulation tool.

## 2. Results and Discussion

### 2.1. Process Results

The biodiesel production capacities for all three processes using acid, base and lipase as catalysts are 8 kiloton/year. In general, WCO contains around 2% to 7% free fatty acids (FFA) and therefore, it is assumed in this study that the WCO contains 6% FFA [15]. The reaction conditions of all the three catalysts are kept in optimum conditions, such as the reaction temperature at 50 °C for the lipase and at 65 °C for the acid and base catalysts. Molar ratio of oil to methanol was 1:4. The reaction time was 6 h for chemical and enzymatic reactions. Although the reaction time for biodiesel production using base catalyst is usually shorter than 6 h, it is assumed that the reaction time for all of the chemical and enzymatic reactions in this study was 6 h to achieve higher biodiesel conversions and equal biodiesel production capacities for all processes (8 kiloton/year). In addition, 5 wt % of chemical catalysts (acid/base) and 10 wt % of enzyme were used for the reactions. In this optimum reaction conditions, biodiesel conversions for all three catalysts are above 90% [16,17,18]. In addition, Novozym-435 catalyzed biodiesel can be recycled for 200 times without obvious decrease in biodiesel yield [18]. Therefore, it is assumed that the biodiesel yield in this study is 100% and the lipase reusability is 200 times. The acid, base and lipase catalyzed biodiesel production processes were analyzed in accordance with the optimum conditions. The obtained results of each individual process are presented in Table 1.

**Table 1:** Operating conditions and simulation results using chemical and enzyme catalysts.

Components	Catalyst		
	Base	Acid	Enzyme
Reaction temperature (°C)	65	65	50
Molar ratio (oil:methanol = 1:x)	4	4	4
Lipase recyclability (times)	-	-	200
Feed streams per day			
Waste cooking oil (L)	26,848.90	26,848.90	26,848.90
Catalyst (kg)	1,206.72	1,206.72	12.07
Methanol (L)	4,339.70	4,339.70	4,339.70
Product streams per day			
Biodiesel (100%) (kg)	24,242.42	24,242.42	24,242.42
Glycerol (kg)	2,469.18	2,469.18	2,469.18
Biodiesel conversion (%)	100.00	100.00	100.00

### 2.2. Economic Assessment

Several assumptions were made for the techno-economic assessment. The equipment cost, raw material cost, biodiesel and glycerol prices are estimated in accordance with the reported processes [13,18,19,20,21]. According to these reports, the total equipment costs (TEC) for the biodiesel production process for each individual catalyst are calculated (Table 2).

**Table 2:** Total equipment costs (TEC).

Equipment	Amount			Unit Cost (USD)	Total Cost (USD)		
	Base	Acid	Enzyme		Base	Acid	Enzyme
Tank (100 m^3^)	7	6	6	$66,478.00	$465,346.00	$398,868.00	$398,868.00
Splitter, mixer (double-arm sigma) (15 kW)	4	2	1	$56,550.00	$226,200.00	$113,100.00	$56,550.00
Reactor (15 m^3^)	5	4	1	$88,906.00	$444,530.00	$355,624.00	$88,906.00
Separator (decanter) (bottom driven 0.6 m diameter)	4	3	2	$13,769.00	$55,076.00	$41,307.00	$27,538.00
Extraction column, distillation column (1 m diameter, 15 m height)	4	4	1	$169,971.00	$679,884.00	$679,884.00	$169,971.00
				TEC	$1,871,036.00	$1,588,783.00	$741,833.00

**Scheme 1:**
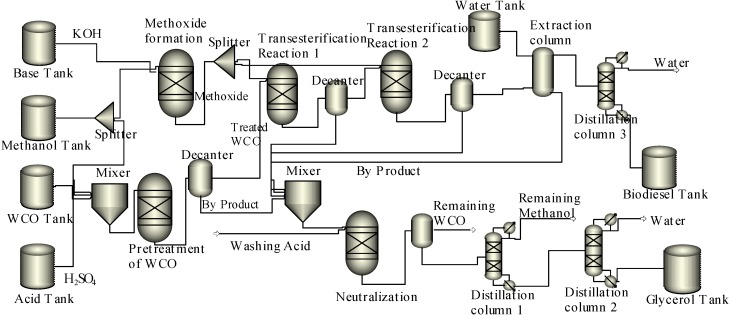
Biodiesel production process using base catalyst.

**Scheme 2:**
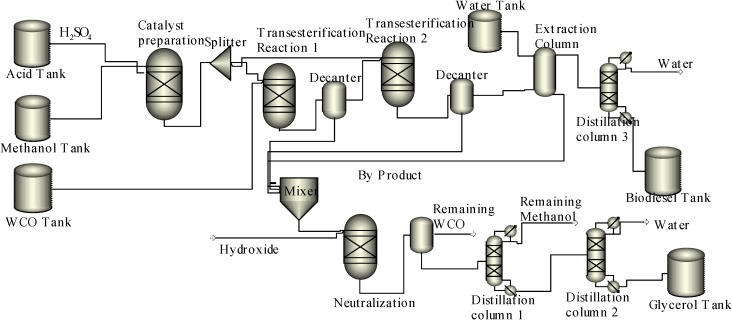
Biodiesel production process using acid catalyst.

**Scheme 3:**
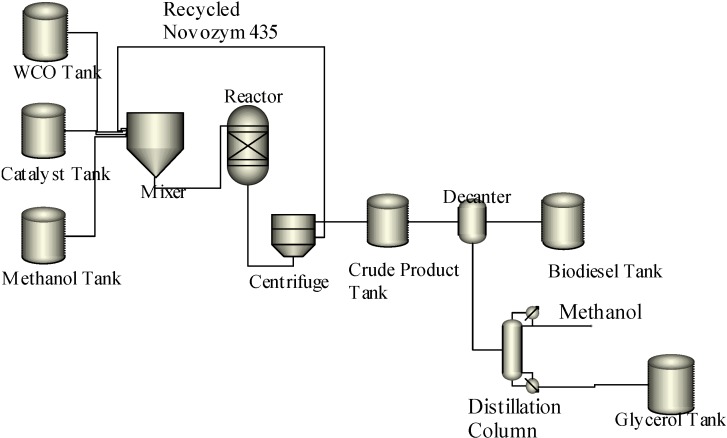
Biodiesel production process with enzyme catalyst.

In Table 2, the amount of each specific equipment used for the biodiesel production processes with different catalysts was determined from the process flow diagrams of biodiesel production (Scheme 1, Scheme 2 and Scheme 3). Meanwhile, the size of each piece of equipment was calculated and approximated from the biodiesel production capacities per batch. It is assumed in this study that the plant working days are 330 days, the working time is 15 h per day, and two biodiesel batches are produced each day.

The capital investment cost (CIC) and specific investment cost (SIC) for each biodiesel production process using different catalysts are estimated. The capital investment cost (CIC) is the sum of total capital cost (TCC) and working capital, while the specific investment cost (SIC) is the CIC divided by the biodiesel production capacity per year. The CIC and SIC are calculated with respect to the TEC. There are several categories involved in calculating the CIC and SIC [4]. The CIC and SIC for each catalyst are calculated (Table 3).

**Table 3:** Capital investment cost (CIC).

Capital Investment Category	Percentage of TEC (%)	Cost (USD)		
		Base	Acid	Enzyme
Total equipment cost (TEC)	100	$1,871,036.00	$1,588,783.00	$741,833.00
Equipment delivery cost	10	$187,103.60	$158,878.30	$74,183.30
Installation cost	20	$374,207.20	$317,756.60	$148,366.60
Instrumentation & control	10	$187,103.60	$158,878.30	$74,183.30
Piping	20	$374,207.20	$317,756.60	$148,366.60
Electrical system	15	$280,655.40	$238,317.45	$111,274.95
Buildings	15	$280,655.40	$238,317.45	$111,274.95
Service facilities	25	$467,759.00	$397,195.75	$185,458.25
Land acquisition	10	$187,103.60	$158,878.30	$74,183.30
Yard improvement	10	$187,103.60	$158,878.30	$74,183.30
Engineer training	30	$561,310.80	$476,634.90	$222,549.90
Legal expenses	10	$187,103.60	$158,878.30	$74,183.30
Contingency	15	$280,655.40	$238,317.45	$111,274.95
	Total capital cost (TCC)	$5,426,004.40	$4,607,470.70	$2,151,315.70
	Working capital	25% of TCC	25% of TCC	25% of TCC
	Capital investment cost (CIC)	$6,782,505.50	$5,759,338.38	$2,689,144.63
	Specific investment cost (SIC)	$847.81	$719.92	$336.14

**Figure 1:**
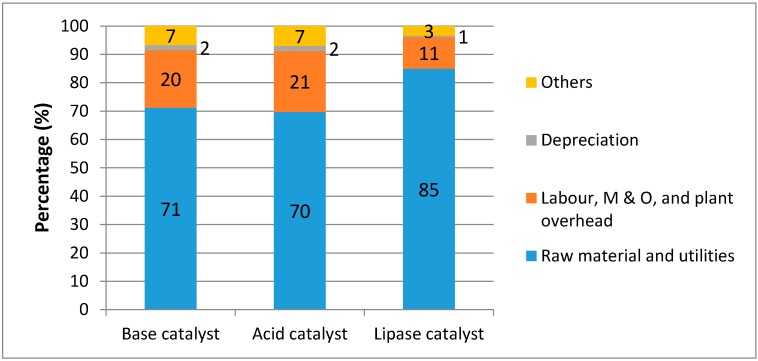
Biodiesel production cost (BPC) breakdown for each catalyst with waste cooking oil (WCO) as a feedstock.

The biodiesel production cost (BPC) is estimated. There are several categories included in the calculations of BPC [4]. The biodiesel production cost for each catalyst is shown in Table 4. The breakdown figure for each category of the BPC with WCO as a feedstock for each catalyst is shown in Figure 1. The raw material and utilities for acid and base catalysts only contributes for 70%–71% of total costs; while in the case for lipase it contributes for 85% of total costs (Figure 1). The raw material and utilities cost while using vegetable oil as a feedstock contributes for around 90% of the total costs [5]. This indicates that utilization of WCO as a low cost feedstock reduced the production cost of biodiesel.

**Table 4:** Biodiesel production cost (BPC).

Category	Unit Cost (USD)	Cost (USD)		
		Base	Acid	Lipase
Raw material cost	$0.57 (base), $0.48 (acid), $0.88 (enzyme)/L WCO	$5,008,424.12	$4,250,217.89	$7,098,606.93
Glycerol revenues	$160.00/ton	$ 130,372.92	$ 130,372.92	$ 130,372.92
Electricity (assuming 60 kWh/ton of biodiesel produced for base catalyst, 55 kWh/ton for acid catalyst, and 40 kWh/ton for lipase)	$0.15/kWh	$72,000.00	$66,000.00	$48,000.00
Labor (assuming 15 employees for base catalyst, 14 employees for acid catalyst, and 12 employees for lipase)	$50,000/employee/year	$750,000.00	$700,000.00	$600,000.00
Maintenance and operational costs (M & O)	10% of TEC	$187,103.60	$158,878.30	$74,183.30
Plant overhead costs	50% of labor and M & O	$468,551.80	$429,439.15	$337,091.65
Depreciation	Straight-line depreciation over 15-year factory life	$124,735.73	$105,918.87	$49,455.53
General expenses	25% of labor and M & O	$234,275.90	$214,719.58	$168,545.83
Property insurance costs	5% of TEC	$93,551.80	$79,439.15	$37,091.65
Contingency	10% of labor, M & O, and plant overhead costs	$140,565.54	$128,831.75	$101,127.50
	Biodiesel production cost (BPC)	$6,948,835.57	$6,003,071.75	$8,383,729.46
	Biodiesel production cost/ton	$868.60	$750.38	$1,047.97

WCO: Waste cooking oil; M & O: Maintenance and operational.

### 2.3. Sensitivity Analysis

**Figure 2:**
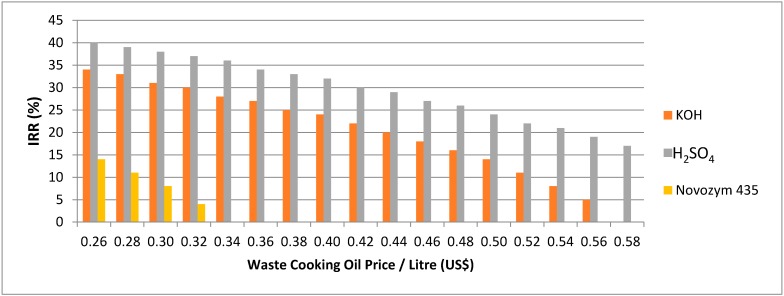
Internal rate of return (IRR) sensitivity analysis with WCO price variation.

**Figure 3:**
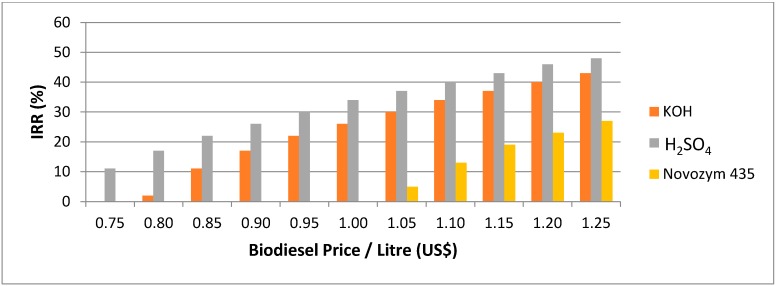
IRR sensitivity analysis with biodiesel price variation.

In the sensitivity analysis, the prices of WCO and biodiesel are varied to determine the correlation of the prices with the internal rate of return (IRR). The minimum IRR for a new investment in an established market is 16% [4]. The first three years are the capital investments and the first-year production has only 50% of the full production amount capacity, while the 4th years onwards rose to 100% of total production capacity. There is a 25% taxation of the gross profit and annual increase of 7% of biodiesel production cost and biodiesel selling price. For the WCO price variation, the reference biodiesel price is $1.01/L (the biodiesel price with vegetable oil as feedstock). The IRR sensitivity analysis for the WCO price variation for each catalyst is shown in Figure 2. For the biodiesel price variation, the reference WCO price in Hong Kong is $0.36/L [13]. The IRR sensitivity analysis for the biodiesel price variation for acid, base and lipase is shown in Figure 3.

Acid catalyst was found to be the most cost-effective for the biodiesel production in Hong Kong (Figure 2 and Figure 3). The acceptable WCO price for the acid catalyst is below $0.58, and the acceptable biodiesel price is above $0.80 (Figure 2 and Figure 3). On the other hand, the acceptable WCO price for the base catalyst is below $0.48, and base catalyzed process and enzyme catalyzed process have acceptable biodiesel price above $0.90 and above $1.15 respectively (Figure 2 and Figure 3). This means that biodiesel production processes using acid and base catalysts can adapt with the variation in the WCO and biodiesel prices.

## 3. Process Design and Case Study

### 3.1. Background of the Case Study

Hong Kong is a small city with a total area of 1104 km^2^. In 2010 Hong Kong’s diesel demand was 1408 kiloton/year [22,23]. It is estimated that the diesel consumption in 2014 is approximately 776 kiloton/year in Hong Kong [23]. The diesel price had been increasing from 2004 to 2012 and the diesel price in 2012 was $1.6/L [24]. The Hong Kong Government has implemented a mandatory policy to use 1%–5% of biodiesel as a blend in petroleum diesel [25]. Due to low mandatory biodiesel blending requirement, a small biodiesel production plant with a production capacity of 8 kiloton/year of biodiesel should be sufficient to produce the required amount of biodiesel for the overall consumption in Hong Kong. Therefore, this paper focuses on the techno-economic studies for a “biodiesel production plant” with a capacity of 8 kiloton/year.

### 3.2. Biodiesel Production Process Using Chemical Catalysts

For biodiesel production using a base catalyst (KOH), the WCO is pretreated with the methanol and sulphuric acid (H_2_SO_4_) as catalyst. Then, remaining methanol is reacted with a base catalyst (KOH) to produce methoxide. In the pretreatment process, esterification reaction is performed to convert the free fatty acid in the WCO into fatty acid methyl ester or biodiesel [26]. The obtained mixture after pretreatment is transferred into a decanter to separate the treated WCO and the water by product. The treated WCO is then transferred into the first reactor, in which the treated WCO is transesterified with methoxide. Subsequently, the product of this reaction is decanted, and after separation of glycerol the obtained filtrate is transferred into a second reactor. In the second reactor, methoxide is added for the second transesterification process. The reaction mixture of this process is decanted. The byproduct glycerol is filtered, leaving the biodiesel, base catalyst (KOH), and remaining glycerol in the filtrate. A stream of water and the filtrate are transferred into an extraction column.

The remaining methanol, KOH, and remaining glycerol are extracted by water, while the biodiesel is separated. Then the biodiesel is transferred into a distillation column to make it dry by removing the remaining moisture. The obtained pure biodiesel is then stored in the biodiesel tank. On the other hand, all the remaining substances such as WCO, methanol, glycerol, and KOH in the reaction are transferred into a reactor for the neutralization process. After neutralization the remaining WCO is decanted; while the remaining methanol and water are distilled into two separated distillation columns. Then, the pure glycerol is stored in a tank. The schematic base catalyzed biodiesel production process is presented in Scheme 1.

The acid catalyzed biodiesel production process does not follow the same mechanism as the base catalyzed process; and it does not require any pretreatment of WCO. Thus, the methoxide formation reactor is replaced with a catalyst preparation reactor to mix the methanol with acid catalyst (H_2_SO_4_). The biodiesel production process using an acid catalyst is shown in Scheme 2.

### 3.3. Biodiesel Production Process Using Lipase

Lipases are extensively used as an alternative to chemical catalysts [27,28]. The production of biodiesel using lipase as a catalyst involved fewer unit operations as compared to the acid and base catalyzed processes. The WCO is reacted with methanol in the presence of lipase (Novozym-435) to form biodiesel and glycerol. The product of the transesterification reaction is transferred into a centrifuge to separate the lipase from the crude product containing biodiesel and glycerol. The lipase is then recycled for the subsequent transesterification reaction. The obtained reaction mixture is transferred into a decanter to separate the byproduct glycerol and remaining methanol from the biodiesel. The pure biodiesel is stored in the biodiesel tank; while, the glycerol is distilled from the methanol and stored in a glycerol tank. The schematic diagram of the biodiesel production process using lipase is presented in Scheme 3.

## 4. Conclusions

In this study, biodiesel production cost in Hong Kong from WCO as a feedstock is estimated. The use of acid and base catalysts for the biodiesel production are found to be less expensive ($0.80 and $0.90) than the average biodiesel price in Hong Kong ($1.01). Acid catalyzed method is the most cost-effective production process. On the other hand, application of lipase as a catalyst is found to be expensive ($1.15). In addition, production of biodiesel using acid and base as catalysts can withstand variations from the WCO and biodiesel prices.
